# Factors associated with preventive behaviors for microvascular complications and self-reported neuropathy symptoms among community-dwelling individuals with type 2 diabetes in Thailand

**DOI:** 10.1186/s12889-026-28787-y

**Published:** 2026-07-27

**Authors:** Nanda Win, Natthida Phoosuwan, Junjira Phasom, Pakorn Pusuwan, Sandeep Poddar, Pankaew Tantirattanakulchai, Nitikorn Phoosuwan

**Affiliations:** 1 Faculty of Public Health, Thammasat University, 99 Moo18, Khlong Nueng, Khlong Luang, Pathum Thani, Pathum Thani, 12121 Thailand; 2 Pathum Thani Provincial Administrative Organization, Pathum Thani, Thailand; 3 Sakon Nakhon Hospital, Ministry of Public Health, Sakon Nakhon, Thailand; 4 Department of Applied Thai Traditional Medicine, University of Phayao, Phayao, Thailand; 5 Lincoln University College, Selangor, DE Malaysia; 6 Department of Health Education and Behavioral Sciences, Faculty of Public Health, Mahidol University, Bangkok, Thailand; 7 Department of Public Health and Caring Sciences, Uppsala University, Box 564, Uppsala, SE-751 22 Sweden

**Keywords:** Type 2 diabetes mellitus, Microvascular complications, Preventive behaviors, Self-efficacy, Diabetes distress; neuropathy

## Abstract

**Background:**

Type 2 diabetes mellitus (T2DM) is a significant public health issue globally, with a high burden of microvascular complications, including diabetic retinopathy, nephropathy, and neuropathy. Preventive behaviors play a critical role in reducing the risk and progression of these complications; however, the factors influencing such behaviors remain insufficiently understood. This study aimed to examine factors associated with adherence to preventive screening behaviors for microvascular complications and the reporting of neuropathy symptoms among community-dwelling individuals with T2DM in Sakon Nakhon Province, Thailand.

**Methods:**

This cross-sectional study was conducted among 447 individuals with T2DM. Data was collected on sociodemographic characteristics, health-related factors, and psychosocial variables, including diabetes knowledge, self-efficacy, and diabetes distress. Preventive behaviors and self-reported neuropathy symptoms were assessed using structured questionnaires. Simple and multiple linear regression analyses were used to determine factors associated with preventive behaviors and neuropathy symptoms. Statistical significance was defined as a *p*-value of less than 0.05.

**Results:**

The average age of participants was 61.07 ± 9.03 years, and a large proportion were female (77.2%). In multiple linear regression analysis, age (*p* < 0.05) and gender (*p* < 0.05) showed a significant association with eye-related preventive behaviors. Duration of diabetes (*p* < 0.05), knowledge of diabetes (*p* < 0.05), and self-efficacy (*p* < 0.05) were significant predictors of kidney-related preventive behaviors. Additionally, comorbidity (*p* < 0.05), diabetes knowledge (*p* < 0.001) and diabetes distress (*p* < 0.001) showed a significant association with self-reported neuropathy symptoms.

**Conclusion:**

Community-dwelling individuals with T2DM may have limited knowledge of diabetes, particularly those requiring more complex management. Improving knowledge, self-efficacy, and psychological well-being is important to support preventive behaviors and reduce the risk of microvascular complications.

**Supplementary Information:**

The online version contains supplementary material available at https://doi.org/10.1186/s12889-026-28787-y.

## Background

Diabetes is a chronic disorder of metabolism in which prolonged elevations in blood sugar contribute to gradual damage across multiple organ systems, including the heart, kidneys, eyes, nerves, and blood vessels [1]. It is among the most prevalent metabolic disorders worldwide, with its prevalence among adults having increased markedly over recent decades [2].

Among the different forms of diabetes, T2DM is the most encountered, typically occurring in adults, and is characterized by insulin resistance and/or insufficient insulin production. It makes up approximately 90–95% of all diabetes cases and is recognized as an escalating global public health problem. Over the past 30 years, global diabetes prevalence has surged across all income levels due to aging populations, urbanization, physical inactivity, and shifting diets. Currently, an estimated 830 million people live with diabetes predominantly in low- and middle-income countries where both total cases and untreated rates continue to rise steadily. Compounding this, a slow clinical progression creates a 4–7 year asymptomatic window, leaving 30–50% of individuals undiagnosed until clinical complications surface [3].

Among these, microvascular complications are highly aggressive, occurring more frequently and rapidly than macrovascular issues, with diabetes elevating microvascular disease risk 10- to 20-fold. Epidemiological data show that 18–30% of patients already present with at least one microvascular complication at initial diagnosis [4], driven largely by persistent, untreated hyperglycemia [5]. In Thailand, this trend is rising sharply; data from a major tertiary center at Siriraj Hospital reported baseline prevalence rates of 37% for diabetic nephropathy, 31% for diabetic retinopathy, and 40% for diabetic foot conditions [6]. Although early screening and targeted self-care behaviors can effectively delay or prevent these outcomes, clinical data from urban tertiary centers cannot be directly generalized to rural populations. In resource-limited regions like Northeastern Thailand, managing these conditions depends heavily on localized community primary care frameworks, highlighting a critical need to evaluate the behavioral and psychosocial factors that dictate patient-led preventive habits.

Although broad trends in Thai diabetes care can be observed through national data, striking regional inequities remain; specifically, the country’s Northeastern province bears a disproportionate burden of health system dropouts and gaps in care along the clinical pathway. As a rural and rapidly aging province in Northeast Thailand, Sakon Nakhon exemplifies a highly vulnerable yet overlooked research setting. The vast majority of community-dwelling older adults managing T2DM in this area face severe deficits in both fundamental disease literacy and self-care competencies [7]. Additionally, structural and geographical challenges further compromise T2DM care in this area. Local residents rely heavily on primary care units at the sub-district level for standard medical visits; consequently, securing essential preventive services like routine eye evaluations or advanced renal surveillance requires either extensive travel to major provincial hospitals or moving through complex institutional referral pathways [7, 8]. Therefore, this study examined factors associated with adherence to preventive screening behaviors for microvascular complications and the reporting of neuropathy symptoms among community-dwelling individuals with T2DM in Sakon Nakhon Province, Thailand.

## Methods

### Study design and setting

This study employed a community-based cross-sectional design carried out in Sakon Nakhon Province, a northeastern region of Thailand, from 1 June to 30 June 2025. Sakon Nakhon is ranked among the ten poorest provinces in the country and comprises a mixture of rural and urban communities. Approximately 80,405 individuals in the province are registered as having T2DM. Participants were recruited from three districts located approximately 20–60 km from Sakon Nakhon city. The study population included individuals diagnosed with T2DM who attended healthcare services at selected Sub-district Health Promoting Hospitals (SHPHs) during the study period.

### Participants

The study sample comprised 447 community-dwelling individuals, including 345 women and 102 men. A multistage random sampling technique was employed to select participants. First, three districts A, B and C were randomly selected from the total of eighteen districts in Sakon Nakhon Province. Second, one SHPH was randomly selected from each of the chosen districts. Third, the names on the T2DM registration lists at each selected SHPH served as the sampling frame. A lottery method was used, where individual identification numbers were drawn at random from a container until the required sample size for each site was achieved.

The sample size was calculated by the researchers (PP, NP and SP) using the formula proposed by Wayne with a confidence level of 95%, a proportion (p) of 0.40, a population size (N) of 80,405, and a margin of error (d) of 0.05, resulting in a minimum required sample size of 368 participants. The sample size was inflated by 20% to account for missing data, resulting in 447 participants included in the analysis. Eligible participants were individuals with T2DM diagnosed for ≥ 1 year, able to read and respond to a Thai questionnaire, and registered at selected SHPHs. Those with diagnosed mental disorders, receiving psychiatric medications, or unwilling to participate were excluded.

### Data collection

Data collection was conducted from three SHPHs located in three districts of Sakon Nakhon Province. NP, NaP and JP asked permission from the selected SHPH’s directors to access individuals with T2DM registered lists at the diabetes clinics and to conduct data collection. Questionnaires were administered to participants who were randomly selected from the registration lists by NP, NaP and JP. The questionnaire was voluntarily completed by each participant at the diabetes clinic, with an average completion time of approximately 20 min. All complete questionnaires were reviewed for completeness and accuracy prior to data analysis.

### Instrument

A structured questionnaire consisting of three parts was developed for this study by the researchers (JP, PP, SP and NP). Part 1 included sociodemographic (age, gender, marital status, education level, employment, comorbidity, current smoker, current drinker). Part 2 included duration of diabetes mellitus, knowledge of diabetes mellitus, self-efficacy, and diabetes distress. Part 3 included self-reported symptoms of diabetic peripheral neuropathy, preventive behavior of diabetic retinopathy and diabetic nephropathy. The questionnaire’s content validity was established by three Thai researchers with doctoral qualifications. All items demonstrated an Index of Item–Objective Congruence (IOC) greater than 0.50, indicating acceptable content validity. Internal consistency was evaluated in a pilot study among 30 individuals with T2DM in one SHPH outside Sakon Nakhon Province. The Cronbach’s alpha coefficient for the parts of (1) preventive behavior of retinopathy, (2) kidney disease screening behaviors, (3) diabetes self-management behaviors, (4) diabetes-related emotional distress and (5) symptoms of diabetic peripheral neuropathy were greater than 0.70, indicating acceptable reliability for data collection.

The Diabetes Knowledge Test (DKT) is a valid and reliable measure of general diabetes knowledge, consisting of 14 items. Each item is presented in a multiple-choice format, with one point awarded for each correct response [9]. This study used the Thai-validated version, with scores ranging from 0 to 14; higher scores represent higher level of knowledge [7].

The preventive behavior of retinopathy questionnaire was used to assess participants’ knowledge related to the prevention of diabetic retinopathy and consisted of five items [10]. Each item is presented in a multiple-choice format, with one point awarded for each correct response. The total possible score ranges from 0 to 5, with higher scores indicating greater knowledge of diabetic retinopathy prevention. This part was translated into Thai and back-translated by bilingual experts before used.

A structured questionnaire consisting of eight items was used to assess kidney disease screening behaviors and related preventive factors for diabetic nephropathy. The instrument included four items on screening practices, three items on perceptions (necessity, accessibility, and cost), and one item on willingness to undergo screening. Responses were recorded using five-point Likert scales. Higher scores indicated more favorable preventive factors [11]. The instrument was translated into Thai and back-translated by bilingual experts and has been previously validated in a Thai population.

The Diabetes Self-Efficacy Scale (DSES) was used to assess participants’ confidence in performing diabetes self-management behaviors. The instrument consists of eight items covering domains such as dietary control, physical activity, blood glucose management, and decision-making regarding medical care. It is a 10-point Likert scale. The overall self-efficacy score was obtained by averaging item responses, with higher scores denoting stronger self-efficacy [12]. The scale has been translated into Thai and back-translated by experts.

The Diabetes Distress Scale (DDS-17) was for determining diabetes-related emotional distress. The instrument consists of 17 items. Each item is rated on a 6-point Likert scale. Total scores range between 17 and 102, with higher scores denoting greater levels of diabetes-related distress [13]. It was translated into Thai and back-translated by experts.

The Michigan Neuropathy Screening Instrument (MNSI) was used to assess self-reported symptoms of diabetic peripheral neuropathy. The MNSI questionnaire consists of 15 items related to common neuropathic symptoms, including numbness, pain, tingling, and sensitivity in the feet. Each item is answered as “yes” or “no,” and responses are scored according to the standard MNSI scoring criteria. The total score is calculated by summing the relevant items, with higher scores indicating a greater likelihood of neuropathy. The Thai-validated MNSI is a widely used and validated screening tool for diabetic neuropathy and has demonstrated good reliability and validity in both clinical and research settings [14].

### Data analysis

Data was analyzed using SPSS version 26 (SPSS Inc., Chicago, IL, USA) by the research team (NW, PT, and NP). Descriptive statistics summarized participant characteristics. Continuous variables were reported as means ± SD, and categorical variables as frequencies and percentages.

Simple linear regression analysis was performed for examining the associations between independent variables (sociodemographic characteristics, clinical factors, knowledge of diabetes mellitus, self-efficacy, and diabetes distress) and outcome variables, including preventive behaviors related to retinopathy, nephropathy, and self-reported neuropathy symptoms.

Categorical independent variables were dummy-coded before being entered into the simple and multiple linear regression models. The reference categories were as follows: male for gender, unmarried for marital status, primary school education or lower for educational level, unemployed for employment status, absence of comorbidity for comorbidity status, non-current smoker for smoking status, and non-current drinker for alcohol consumption.

Multiple linear regression analysis was then conducted for identifying independent factors associated with each outcome variable. Variables associated with the outcome at a significance level of *p* < 0.20 in the univariate analyses were considered candidates for inclusion in the multiple linear regression models. Standardized beta coefficients (β) and corresponding *p*-values were reported to investigate the strength and direction of associations. Assumptions of linear regression, including normality, linearity, homoscedasticity, and absence of multicollinearity, were evaluated before model interpretation. Statistical significance was set at a two-tailed *p* < 0.05.

### Result

A total of 447 individuals with T2DM were included in this study. The mean age of participants was 61.07 ± 9.03 years, ranging from 20 to 88 years. The majority of participants were female (77.2%) and married (73.2%). Most had an education level of primary school or below (81.2%) and were currently employed (89.9%). Regarding health-related characteristics, 71.8% of participants reported having comorbidities. The majority were non-smokers (93.7%) and non-drinkers (77.2%). The mean duration of diabetes was 11.80 ± 8.13 years, with a range of 1 to 58 years. In terms of psychosocial factors, the mean knowledge score of diabetes was 6.47 ± 1.93, self-efficacy score was 55.28 ± 15.29, and diabetes distress score was 26.73 ± 13.22. (Table 1)

**Table 1:** Sociodemographic characteristics of People with Type 2 Diabetes Mellitus in Sakon Nakhon, a Northeastern province in Thailand (*n* = 447)

Characteristics	Frequency	Percentage
Age, years		
Mean ± SD	61.07 ± 9.03	
Range	20–88	
Gender		
Female	345	77.2
Male	102	22.8
Marital status		
Married	327	73.2
Unmarried	120	26.8
Education level		
≤ Primary School	363	81.2
> Primary School	84	18.8
Employment		
Employed	402	89.9
Unemployed	45	10.1
Comorbidity		
Yes	321	71.8
No	126	28.2
Current smoker		
No	419	93.7
Yes	28	6.3
Current drinker		
No	345	77.2
Yes	102	22.8
Duration of diabetes mellitus		
mean ± SD	11.80 ± 8.13	
Range	1–58	
Knowledge of diabetes mellitus		
mean ± SD	6.47 ± 1.93	
Range	0–11	
Self-Efficacy		
mean ± SD	55.28 ± 15.29	
Range	16–80	
Diabetes Distress		
Mean ± SD	26.73 ± 13.22	
Range	17–102	

*SD* Standard Deviation

According to the simple linear regression analysis, older age was significantly associated with lower eye-related preventive behavior scores (standardized β = −0.95, 95% CI: -0.018 to 0.000, *p* = 0.044), whereas female gender was associated with higher eye-related preventive behavior scores (standardized β = 0.108, 95% CI: 0.032 to 0.413, *p* = 0.022). For kidney-related preventive behaviors, a longer duration of diabetes mellitus (β = 0.131, 95% CI: 0.015 to 0.085, *p* = 0.006), greater diabetes knowledge (β = 0.141, 95% CI: 0.079 to 0.376, *p* < 0.01), and higher self-efficacy (β = 0.153, 95% CI: 0.012 to 0.050, *p* < 0.01) were each associated with higher preventive behavior scores. In addition, the presence of comorbidity (β = 0.115, 95% CI: 0.111 to 1.004, *p* = 0.015) and higher diabetes distress (β = 0.227, 95% CI: 0.022 to 0.052, *p* < 0.001) were positively associated with self-reported neuropathy, whereas greater diabetes knowledge was negatively associated with self-reported neuropathy (β = −0.235, 95% CI: −0.366 to − 0.163, *p* < 0.001) (Table 2). In addition, no evidence of multicollinearity was observed among the independent variables. The tolerance values ranged from 0.717 to 0.979, while the variance inflation factor (VIF) values ranged from 1.021 to 1.394, indicating that multicollinearity was not a concern.

**Table 2:** Association between independent variables and preventive behaviors using simple linear regression analysis

Variables	Eye-related preventive behavior scores			Kidney-related preventive behaviors			Self-reported neuropathy		
	Standardized Beta	Adjusted OR (95%CI)	*p*	Standardized Beta	Adjusted OR (95%CI)	*p*	Standardized Beta	Adjusted OR (95%CI)	*p*
Age	-0.95*	-0.018-0.000	0.044	0.033	-0.021-0.043	0.492	0.066	-0.007-0.038	0.164
Gender	0.108*	0.032–0.413	0.022	-0.004	-0.723-0.658	0.926	-0.030	-0.639-0.325	0.523
Marital status	0.079	-0.026-0.335	0.094	-0.041	-0.689-0.619	0.916	0.021	-0.354-0.559	0.659
Education level	0.056	-0.081-0.330	0.233	-0.016	-0.873-0.610	0.728	-0.027	-0.669-0.367	0.567
Employment	-0.091	-0.528-0.004	0.054	0.048	-0.461-1.463	0.306	-0.041	-0.965-0.379	0.391
Comorbidity	-0.062	-0.297-0.059	0.190	0.009	-0.584-0.704	0.855	0.115*	0.111–1.004	0.015
Current smoker	-0.016	-0.389-0.275	0.736	-0.014	-1.373-1.018	0.771	0.058	-0.317-1.351	0.224
Current drinker	0.034	-0.122-0.261	0.475	-0.023	-0.861-0.519	0.627	-0.073	-0.858-0.104	0.125
Duration of diabetes mellitus	0.036	-0.006-0.014	0.455	0.131**	0.015–0.085	0.006	0.054	-0.011-0.039	0.256
Knowledge of diabetes mellitus	-0.001	-0.042-0.041	0.985	0.141**	0.079–0.376	0.003	-0.235***	-0.366 to -0.163	< 0.001
Self-Efficacy	0.0018	-0.15-0.045	0.125	0.153**	0.012–0.050	0.001	-0.018	-0.016-0.011	0.711
Diabetes Distress	0.043	-0.003-0.009	0.361	-0.079	-0.040-0.003	0.096	0.227***	0.022–0.052	< 0.001

*OR* Odd ratio, *CI* Confidence Interval

* *p* < 0.05, ** *p* < 0.01, *** *p* < 0.001

According to the multiple linear regression analysis, older age remained independently associated with lower eye-related preventive behavior scores (standardized β = −0.092, 95% CI: -0.018 to 0.000, *p* < 0.05), whereas female gender remained associated with higher eye-related preventive behavior scores (β = 0.099, 95% CI: 0.030 to 0.412, *p* < 0.05). For kidney-related preventive behaviors, a longer duration of diabetes (β = 0.139, 95% CI: 0.018 to 0.088, *p* = 0.003), greater diabetes knowledge (β = 0.138, 95% CI: 0.076 to 0.372, *p* < 0.05), and higher self-efficacy (β = 0.138, 95% CI: 0.010 to 0.047, *p* < 0.05) were independently associated with higher preventive behavior scores. In addition, the presence of comorbidity (β = 0.101, 95% CI: 0.061 to 0.915, *p* < 0.05) and higher diabetes distress (β = 0.201, 95% CI: -0.018 to 0.048, *p* < 0.001) were positively associated with self-reported neuropathy symptoms, whereas greater diabetes knowledge was negatively associated with self-reported neuropathy symptoms (β = −0.218, 95% CI: -0.345 to -0.146, *p* < 0.001) (Table 3).

**Table 3:** Association between independent variables and preventive behaviors using multiple linear regression analysis

Variables	Standardized Beta	Adjusted OR (95%CI)	*p*
Eye-related preventive behavior scores			
Age	-0.92*	-0.018–0.000	0.048
Gender	0.99*	0.030–0.412	0.034
Kidney-related preventive behaviors			
Duration of diabetes mellitus	0.139**	0.018–0.088	0.003
Knowledge of diabetes mellitus	0.138**	0.076–0.372	0.003
Self-Efficacy	0.138**	0.010–0.047	0.003
Self-reported neuropathy			
Comorbidity	0.101*	0.061–0.915	0.025
Knowledge of diabetes mellitus	-0.218***	-0.345 to -0.146	< 0.001
Diabetes Distress	0.201***	0.018–0.048	< 0.001

*OR* Odd ratio, *CI* Confidence Interval

* *p* < 0.05, ** *p* < 0.01, *** *p* < 0.001

## Discussion

This study examined factors associated with preventive behaviors related to diabetic retinopathy, nephropathy, and self-reported neuropathy symptoms among community-dwelling individuals with T2DM. The findings highlight the multifactorial nature of diabetes self-management, encompassing demographic, clinical, and psychosocial determinants. The present study found that age and gender were significantly associated with eye-related preventive behaviors, both in simple and multiple linear regression analyses. The negative association between age and preventive behaviors observed in this study is consistent with previous literature [15, 16] indicating that older adults was significantly associated with lower utilization rates of regular eye screening services, despite having a higher risk of vision impairment. This may be attributed to age-related barriers such as reduced mobility, limited access to healthcare services, and lower health literacy, which can hinder engagement in appropriate eye care practices. In contrast, gender differences indicate that females may be more likely to adopt preventive behaviors, which is consistent with previous literature suggesting that women are more likely to engage in health-seeking behaviors and adhere to medical advice, which may contribute to better preventive practices [17].

For kidney-related preventive behaviors, a longer duration of diabetes was significantly and positively associated with better preventive practices, indicating that individuals who have lived with the disease longer tend to demonstrate higher engagement in these behaviors. This finding suggests that prolonged exposure to the disease may enhance patients’ experience, awareness, and interaction with healthcare services, thereby contributing to better self-care behaviors, including regular screening and monitoring for kidney complications [18]. Interestingly, this behavioral finding appears to contrast with existing clinical evidence, which demonstrates that longer diabetes duration is strongly associated with progressive renal decline and an increased risk of chronic kidney disease, independent of age and other metabolic factors [19]. This discrepancy highlights a critical distinction between behavioral adaptation and physiological outcomes. While individuals with longer disease duration may adopt better preventive behaviors, cumulative hyperglycemic exposure continues to exert deleterious effects on renal function, which may not be fully reversible through behavioral changes alone. Taken together, these findings suggest that although self-management behaviors may improve over time, they may be initiated too late to prevent long-term microvascular damage. This underscores the importance of early intervention, sustained glycemic control, and timely implementation of nephroprotective strategies from the early stages of diabetes. Future interventions should therefore focus not only on enhancing self-management behaviors but also on promoting early and continuous prevention to mitigate the long-term impact of diabetes duration on kidney outcomes. Importantly, knowledge and self-efficacy were consistently associated with better preventive behaviors, underscoring the critical role of patient education and confidence in self-management. These findings align with health behavior theories, such as Social Cognitive Theory, which emphasize the importance of self-efficacy in influencing health-related behaviors [20, 21].

In terms of self-reported neuropathy symptoms, knowledge of diabetes and diabetes-related distress were identified as significant factors. The negative association between diabetes knowledge and neuropathy symptoms suggests that individuals with a better understanding of diabetes may be more capable of adopting behaviors that prevent or delay the onset of complications. This result was supported by recent evidence that health literacy plays a critical role in diabetes management, as individuals with higher levels of health literacy linked to improved glycemic outcomes and a lower likelihood of complications such as neuropathic symptom [22]. Additionally, diabetes distress showed a significant positive association with self-reported neuropathy symptoms. This suggests that a higher psychological burden may coexist with or exacerbate the perception of somatic symptoms; alternatively, experiencing distressing physical neuropathic symptoms may concurrently elevate a patient’s psychological burden. This result was supported by recent evidence pointing out that higher diabetes distress is linked to poorer adherence behaviors and suboptimal glycemic control, which may contribute to the exacerbation of physical symptoms, including neuropathy. These findings highlight the important role of psychosocial factors in tracking closely with both behavioral and self-reported clinical indicators in diabetes management [23]. In addition, the presence of comorbidities was significantly associated with increased self-reported neuropathy symptoms. This finding suggests that individuals with multiple health conditions may be at greater risk of developing or experiencing more severe neuropathic symptoms.

These outcomes carry direct implications for resource-limited primary care within Thailand’s Universal Health Coverage framework. Because diabetes distress tracks closely with poor compliance and physical symptoms, incorporating brief mental health screenings at SHPHs supported by village health volunteers home assessments could improve early behavioral monitoring. Furthermore, overcoming the lower screening rates found among older adults requires addressing geographic access barriers to specialized diagnostic facilities. Policymakers should focus on deploying SHPH-coordinated mobile screening services, including mobile fundus photography and decentralized microalbuminuria testing, to deliver essential preventive interventions directly to rural communities. This study has certain limitations. Causal relationships cannot be inferred due to the cross-sectional study design, and self-reported measures, particularly for neuropathy symptoms, may be subject to reporting bias. Finally, the study population was drawn from a specific geographic setting, which may limit the generalizability of the findings to other populations.

## Conclusion

In conclusion, preventive screening behaviors and self-reported neuropathy symptoms among community-dwelling individuals with T2DM are shaped by a distinct combination of demographic, clinical, and psychosocial factors. Rather than focusing solely on biomedical tracking, public health interventions in rural settings must focus on mitigating diabetes distress, enhancing self-efficacy, and improving disease literacy. Addressing these psychosocial and educational determinants is essential to optimize patient adherence to essential eye, kidney, and foot screening regimens within primary care frameworks.

## Supplementary Information

Supplementary Material 1.

## Data Availability

All data generated or analyzed during this study are included in this published article and its supplementary information files.
