# Supplementary material for: Factors associated with preventive behaviors for microvascular complications and self-reported neuropathy symptoms among community-dwelling individuals with type 2 diabetes in Thailand
Source: BMC Public Health. 2026 Jul 27;26:3034. doi: 10.1186/s12889-026-28787-y (PMC13688728; doi:10.1186/s12889-026-28787-y)
Supplement: Supplementary file 1 [file 12889_2026_28787_MOESM1_ESM.docx]

**Diabetes Knowledge Test**

**Instructions: Please circle on the answer you believe is correct.**

1. Which of the following best describes a diabetic diet?

- A. Common food consumed by Thai people
- B. Healthy food choices for most people
- C. Too high-carbohydrate food consumed by most people
- D. Too high-protein food consumed by most people

2. Which of the following is highest in carbohydrate?

- A. Chicken
- B. Cheese
- C. Nut products
- D. Bread

3. Which of the following foods is high in fat?

- A. Low fat milk
- B. Orange juice
- C. Corn
- D. Honey

4. Which of the following is a “free food?”

- A. Any unsweetened food
- B. Any food that has “fat free” on the label
- C. Any food that has “sugar free” on the label
- D. Any food providing less than 20 calories per serving

5. HbA1c is a measure of your average blood glucose level for the past:

- A. day
- B. week
- C. 6-12 weeks
- D. 6 months

6. What is the best method for monitoring blood glucose levels at home?

- A. Urine testing
- B. Fingertip blood glucose test (Capillary blood glucose)
- C. Both methods are equally effective

7. How do unsweetened fruits (no added sugar) affect blood sugar levels?

- A. Decrease blood sugar levels
- B. Increase blood sugar levels
- C. No effect on blood sugar levels

8. Which of the following should NOT be done if a person has low blood sugar (hypoglycemia)?

- A. Take 3 pieces of candy
- B. Drink half a glass of orange juice
- C. Drink one can of sugar-free soda
- D. Drink one glass of skim milk

9. For those who manage themselves well, what is the effect of regular exercise on blood sugar levels?

- A. Decreases blood sugar levels
- B. Increases blood sugar levels
- C. No effect on blood sugar levels

10. How does an infection affect blood sugar levels?

- A. Decreases blood sugar levels
- B. Increases blood sugar levels
- C. No effect on blood sugar levels

11. What is the best way to care for your feet?

- A. Inspect and wash feet daily
- B. Massage feet with alcohol daily
- C. Soak feet for 1 hour every day
- D. Wear shoes that are larger than your actual size

12. Eating a low-fat diet helps reduce the risk of which disease?

- A. Neurological disease
- B. Kidney disease
- C. Heart disease
- D. Eye disease

13. Numbness and tingling (pins and needles sensation) may be signs of:

- A. Kidney disease
- B. Neuropathy (Nervous system disorder)
- C. Eye disease
- D. Heart disease

14. Which of the following is NOT typically associated with diabetes complications?

- A. Eye problems
- B. Kidney problems
- C. Nervous system problems
- D. Lung problems

**Kidney Complication Prevention Behaviors and Awareness**

**Instructions:** Please mark in the box [ ] by selecting the answer that best matches your actual situation.

1. Have you had your blood sugar levels measured (e.g., finger-prick test) in the past 6 months?

- [ ] Yes
- [ ] No

2. Have you had your HbA1c (accumulated blood sugar level) measured in the past 6 months?

- [ ] Yes
- [ ] No

3. Have you ever had a kidney function test via urine analysis?

- [ ] Yes, at least once a year
- [ ] Yes, but not every year
- [ ] Never
- [ ] Not sure

4. Have you ever had an Estimated Glomerular Filtration Rate (eGFR) test?

- [ ] Yes, at least once a year
- [ ] Yes, but not every year
- [ ] Never
- [ ] Not sure

5. Do you think it is necessary to have kidney function and filtration tests performed annually?

- [ ] Not at all necessary
- [ ] Not very necessary
- [ ] Somewhat necessary
- [ ] Necessary
- [ ] Extremely necessary

6. Do you think it is easy for diabetic patients to access kidney disease screening services?

- [ ] Not easy at all
- [ ] Not very easy
- [ ] Somewhat easy
- [ ] Easy
- [ ] Very easy

7. Do you think the cost of kidney disease screening for diabetic patients (approximately 233 THB) is expensive?

- [ ] Very expensive
- [ ] Somewhat expensive
- [ ] Fair/Appropriate price
- [ ] Somewhat cheap
- [ ] Very cheap

8. Are you willing to undergo annual kidney disease screening provided by the government?

- [ ] Definitely not willing
- [ ] Not very willing
- [ ] Somewhat willing
- [ ] Willing
- [ ] Extremely willing

**The Preventive Behavior of Retinopathy Questionnaire**

1. Is physical exercise important for controlling diabetes?

- [ ] Yes
- [ ] No
- [ ] Don't Know

2. Can diabetes affect your eyes?

- [ ] Yes
- [ ] No
- [ ] Don't Know

3. What is the treatment for diabetic eye disease?

- [ ] None/ I do not know
- [ ] Injections / Surgery / Laser / Combination

4. How often would you check your blood sugar?

- [ ] At least once a month
- [ ] At least once a year

5. Would you worry if you forget to inject insulin/ take tablets?

- [ ] Yes
- [ ] No
- [ ] I do not know

**Part 6: Self-Efficacy (Stanford Patient Education Research Center)**

**Instructions:** Please read each question carefully and mark ✓ in the box that best reflects your level of self-confidence. Choose only one answer per item.

- 1 = Not confident at all
- 10 = Totally confident

| **ข้อมูล** | **ระดับความมั่นใจ**  **(ไม่มั่นใจเลย ไปจนถึง มั่นใจมากที่สุด)** | | | | | | | | | |
| --- | --- | --- | --- | --- | --- | --- | --- | --- | --- | --- |
|  | 1 | 2 | 3 | 4 | 5 | 6 | 7 | 8 | 9 | 10 |
| 1. How confident do you feel that you can eat your meals every 4 to 5 hours every day, including breakfast every day? |  |  |  |  |  |  |  |  |  |  |
| 2. How confident do you feel that you can follow your diet when you have to prepare or share food with other people who do not have diabetes? |  |  |  |  |  |  |  |  |  |  |
| 3. How confident do you feel that you can choose the appropriate foods to eat when you are hungry (for example, snacks)? |  |  |  |  |  |  |  |  |  |  |
| 4. How confident do you feel that you can exercise 15 to 30 minutes, 4 to 5 times a week? |  |  |  |  |  |  |  |  |  |  |
| 5. How confident do you feel that you can do something to prevent your blood sugar level from dropping when you exercise? |  |  |  |  |  |  |  |  |  |  |
| 6. How confident do you feel that you know what to do when your blood sugar level goes higher or lower than it should be? |  |  |  |  |  |  |  |  |  |  |
| 7. How confident do you feel that you can judge when the changes in your illness mean you should visit the doctor? |  |  |  |  |  |  |  |  |  |  |
| 8. How confident do you feel that you can control your diabetes so that it does not interfere with the things you want to do? |  |  |  |  |  |  |  |  |  |  |

**The Diabetes Distress Scale (DDS-17)**

Directions: Living with diabetes can sometimes be tough. There may be many problems and hassles concerning diabetes and they can vary greatly in severity. Problems may range from minor hassles to major life difficulties. Listed below are 17 potential problem areas that people with diabetes may experience. Consider the degree to which each of the 17 items may have distressed or bothered you DURING THE PAST MONTH and circle the appropriate number.

Please note Circle the number gives the best answer for you and please provide an answer for each question. If you feel that a particular item is not a bother or a problem for you, you would circle “1”. If it is very bothersome to you, you might circle “6”

1 = Not a problem

2= A slight problem

3= A moderate problem

4= Somewhat serious problem

5=A serious problem

6= A very serious problem

| **No** | **Items** | **1** | **2** | **3** | **4** | **5** | **6** |
| --- | --- | --- | --- | --- | --- | --- | --- |
| 1 | Feeling the diabetes is taking up too much of my mental and physical energy every day |  |  |  |  |  |  |
| 2 | Feeling that my doctor doesn’t know enough about diabetes and diabetes care |  |  |  |  |  |  |
| 3 | Feeling angry, scared and/or depressed when I think about living with diabetes |  |  |  |  |  |  |
| 4 | Feeling that my doctor doesn’t give me clear enough directions on how to manage my diabetes |  |  |  |  |  |  |
| 5 | Feeling that I am not testing my blood sugars frequently enough |  |  |  |  |  |  |
| 6 | Feeling that I am often failing with my diabetes routine |  |  |  |  |  |  |
| 7 | Feeling that friends or family are not supportive enough of self-care efforts (e.g. planning activities that conflict with my schedule, encouraging me to eat the “wrong” foods) |  |  |  |  |  |  |
| 8 | Feeling that diabetes controls my life |  |  |  |  |  |  |
| 9 | Feeling that my doctor doesn’t take my concerns seriously enough |  |  |  |  |  |  |
| 10 | Not feeling confident in my day-to-day ability to manage diabetes |  |  |  |  |  |  |
| 11 | Feeling that I will end up with serious long-term complications, no matter what I do |  |  |  |  |  |  |
| 12 | Feeling that I am not sticking closely enough to a good meal plan |  |  |  |  |  |  |
| 13 | Feeling that friends or family don’t appreciate how difficult living with diabetes can be |  |  |  |  |  |  |
| 14 | Feeling overwhelmed by the demands of living with diabetes |  |  |  |  |  |  |
| 15 | Feeling that I don’t have a doctor, who I can see regularly enough about my diabetes |  |  |  |  |  |  |
| 16 | Not feeling motivated to keep up my diabetes self management |  |  |  |  |  |  |
| 17 | Feeling that friends or family don’t give me the emotional support that I would like |  |  |  |  |  |  |

**The Michigan Neuropathy Screening Instrument (MNSI)**

**Instructions:** Please take about 2-3 minutes to provide information regarding the sensations in your legs and feet. Select the answer that best matches your symptoms and feelings.

1. Are your legs and/or feet numb?

 Yes	 No

2. Do you ever have any burning pain in your legs and/or feet?

 Yes	 No

3. Are your feet too sensitive to touch?

 Yes	 No

4. Do you get muscle cramps in your legs and/or feet?

 Yes	 No

5. Do you ever have any prickling feelings in your legs or feet?

 Yes	 No

6. Does it hurt when the bed covers touch your skin?

 Yes	 No

7. When you get into the tub or shower, are you able to tell the hot water from the cold water?

 Yes	 No

8. Have you ever had an open sore on your foot?

 Yes	 No

9. Has your doctor ever told you that you have diabetic neuropath

 Yes	 No

10. Do you feel weak all over most of the time?

 Yes	 No

11. Are your symptoms worse at night?

 Yes	 No

12. Do your legs hurt when you walk?

 Yes	 No

13. Are you able to sense your feet when you walk?

 Yes	 No

14. Is the skin on your feet so dry that it cracks open?

 Yes	 No

15. Have you ever had an amputation?

 Yes	 No
